# Overexpression of *AcEXPA23* Promotes Lateral Root Development in Kiwifruit

**DOI:** 10.3390/ijms23148026

**Published:** 2022-07-21

**Authors:** Zhiyong Wu, Ming Li, Yunpeng Zhong, Lan Li, Dawei Cheng, Hong Gu, Xizhi Guo, Xiujuan Qi, Jinyong Chen

**Affiliations:** Key Laboratory of Fruit Breeding Technology of Ministry of Agriculture and Rural Affairs, Zhengzhou Fruit Research Institute, Chinese Academy of Agricultural Sciences, Zhengzhou 450009, China; 82101195086@caas.cn (Z.W.); liming07@caas.cn (M.L.); zhongyunpeng@caas.cn (Y.Z.); lilan01@caas.cn (L.L.); chengdawei@caas.cn (D.C.); guhong@caas.cn (H.G.); guoxizhi@caas.cn (X.G.)

**Keywords:** kiwifruit, expansin, lateral root

## Abstract

Kiwifruit is loved by consumers for its unique taste and rich vitamin C content. Kiwifruit are very sensitive to adverse soil environments owing to fleshy and shallow roots, which limits the uptake of water and nutrients into the root system, resulting in low yield and poor fruit quality. Lateral roots are the key organs for plants to absorb water and nutrients. Improving water and fertilizer use efficiency by promoting lateral root development is a feasible method to improve yield and quality. Expansin proteins plays a major role in lateral root growth; hence, it is important to identify expansin protein family members, screen key genes, and explore gene function in root development. In this study, 41 expansin genes were identified based on the genome of kiwifruit (‘Hongyang’, *Actinidia chinensis*). By clustering with the *Arabidopsis thaliana* expansin protein family, the 41 AcExpansin proteins were divided into four subfamilies. The AcExpansin protein family was further analysed by bioinformatics methods and was shown to be evolutionarily diverse and conserved at the DNA and protein levels. Based on previous transcriptome data and quantitative real-time PCR assays, we screened the candidate gene *AcEXPA23*. Overexpression of *AcEXPA23* in kiwifruit increased the number of kiwifruit lateral roots.

## 1. Introduction

*Actinidia* L., belonging to the family Actinidiaceae, comprises a total of 75 taxa, including 54 species and 21 varieties [1]. Cultivars in production are mainly derived from the original variety of *Actinidia chinensis* Planch. var. *chinensis*, *A. chinensis* Planch. var. *deliciosa (*A. Chev.), and *Actinidia arguta* [2]. Kiwifruit is known as the “king of fruits” because of its unique taste and rich vitamin C content and is popular among consumers [2,3,4]. As of 2019, the kiwifruit planting area in China reached 250,000 ha, far exceeding the sum of the planting areas of other countries in the world and increasing annually [5]. Although the kiwifruit harvested area and output of China rank first in the world, the output per unit area ranks only 20th in the world, with clear import and export trade deficits [5]. Fruit yield and quality are closely related to the efficiency of water and nutrient uptake by plant roots. The root system of kiwifruit is composed of fleshy roots, is mainly distributed in the upper layer of the soil, and is easily affected by the surrounding soil environment [1]. To a certain extent, this limits the moisture and nutrient absorption efficiency of kiwifruit roots, resulting in low fruit production and poor quality [6].

Lateral roots not only promote water absorption and the efficiency with which plants obtain nutrients from the surrounding soil but also provide sufficient mechanical support for the aboveground parts of plants [7]. Lateral root formation is divided into five stages: pre-branch site priming, lateral root initiation, lateral root patterning, lateral root emergence, and lateral root elongation [8,9]. Currently, four models have been identified that regulate lateral root initiation and patterning in *Arabidopsis thaliana*: (i) the IAA28-ARF module [10]; (ii) the SOLITARY-ROOT/IAA14-ARF7-ARF19 module [11,12,13,14]; (iii) the BODENLOS/IAA12-MONOPTEROS/ARF5 module [15,16]; and (iv) the SHY2/IAA3-ARF module [17]. LATERAL ORGAN BOUNDARIES DOMAIN/ASYMMETRIC LEAVES2-LIKE (LBD/ASL) proteins play an important role in the development of lateral organs in plants, including lateral root formation [18]. ARF7 and ARF19 regulate lateral root formation via activation of LBD16/ASL18 and LBD29/ASL16 [14]. LBD18 regulates lateral root formation in conjunction with LBD16 downstream of ARF7 and ARF19 [19,20]. LBD18/ASL20 acts as a specific DNA-binding transcriptional activator that directly regulates EXPANSIN14 (*EXP14*), a gene encoding a cell wall-loosening factor that promotes lateral root emergence in *A. thaliana* [18].

Expansins are cell-wall-loosening proteins that directly induce the extension of the cell wall by disrupting non-covalent bonding between cellulose microfibrils and associated matrix polysaccharides in the cell wall [21,22,23]. Expansin is encoded by a multigenic superfamily in plants divided into four subfamilies: EXPA, α-expansin; EXPB, β-expansin; EXLA, expansin-like A; and EXLB, expansin-like B [24]. Expansins have been shown to be involved in different plant developmental processes, including root development and root hair initiation [25,26], stem internode elongation [27], leaf development [28,29], flower development [30], fruit development and ripening [31], seed germination [32], stomatal movement [33], organ abscission [34], and biotic/abiotic stress response [35,36,37,38].

Our previous study showed that exogenous brassinosteroid (BR) treatment in kiwifruit did significantly promote lateral root development [39]. For in-depth research, we focused, herein, on the expansin gene, the most downstream regulatory gene in current lateral root research. We performed stable genetic transformation of the selected candidate gene *AcEXPA23* in kiwifruit. Our results showed that overexpression of *AcEXPA23* significantly promoted the increase in lateral roots in kiwifruit. This is important for kiwifruit to absorb more water and nutrients through the lateral roots to improve yield and quality.

## 2. Results

### 2.1. Phylogenetic Tree Analysis of the AcExpansin Protein Family

**Figure 1:**
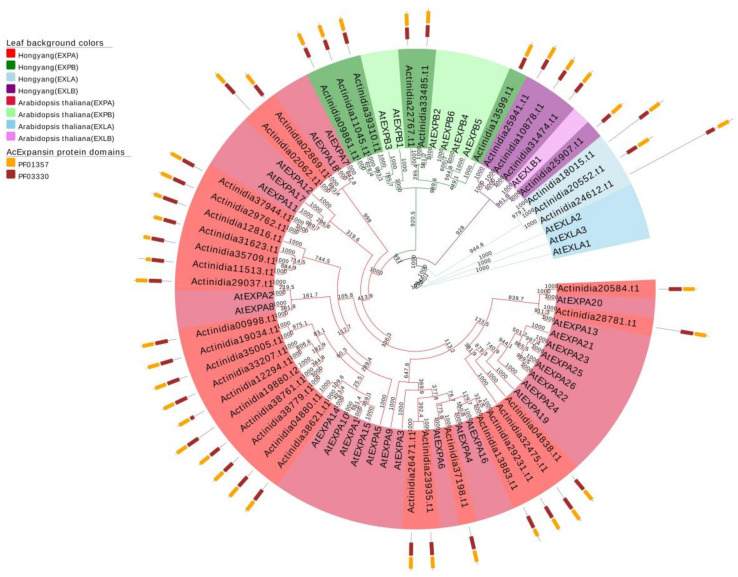
Phylogenetic tree of expansins in kiwifruit and *Arabidopsis*. The phylogenetic tree was constructed using the neighbour-joining method in MEGA11 (related parameter settings: bootstrap: 1000, model/method: p-distance, gaps/missing data treatment: partial deletion). The four expansin subfamilies are: EXPA, α-expansin; EXPB, β-expansin; EXLA, expansin-like A; and EXLB, expansin-like B.

We identified 41 expansin protein family members in kiwifruit. To further classify the expansin gene in kiwifruit, a phylogenetic tree was constructed using 41 AcExpansin protein sequences and with all 36 Arabidopsis expansin protein sequences as references (Figure 1, Table S1). The results showed that the AcExpansin protein family was divided into four subfamilies: AcEXPA, AcEXPB, AcEXLA, and AcEXLB. According to the classification results, the identified AcExpansin protein family members were renamed (Table 1). The subfamily AcEXPA contained 28 genes (*AcEXPA1*–*AcEXPA*28), subfamily AcEXPB contained 6 genes (*AcEXPB1*–*AcEXPB6*), subfamily AcEXLA had 3 genes (*AcEXLA1*–*AcEXLA3*), and subfamily AcEXLB had 4 genes (*AcEXLB1*–*AcEXLB4*).

### 2.2. Physicochemical Properties of AcExpansin Protein Family

We further analysed the physicochemical properties of the 41 AcExpansins. As shown in Table 1, the length of the AcExpansin proteins ranged from 193 to 783 aa. The molecular weight and theoretical pI of the identified AcExpansin proteins ranged from 20.73 kDa to 86.02 kDa and 4.73 to 10.16, respectively. The average isoelectric point was 8.37 and 80% of the expansin proteins had isoelectric points greater than 8.00, indicating that most of the expansin proteins were alkaline. The expansin protein instability index was 22.97–46.03. The lipid solubility index of expansin proteins was 57.67–83.11, with an average value of 70.66. Among them, the lipid solubility index of five AcExpansin proteins exceeded 80.00, indicating that they belonged to the class of thermophilic proteins. The total average hydrophobicity of the 41 AcExpansin genes was −0.441–0.131, indicating they belonged to amphiprotic proteins with comparable hydrophobicity (>0 for hydrophobicity, <0 for hydrophilicity, and ±0.5 for amphiprotic proteins).

### 2.3. AcExpansin Protein Family Chromosomal Location

**Figure 2:**
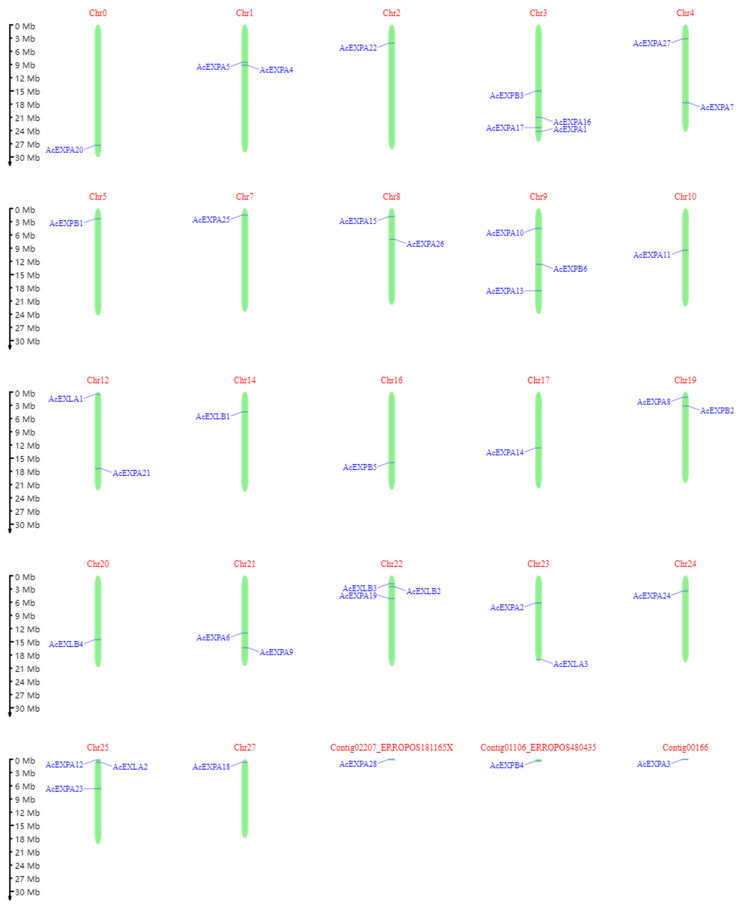
Schematic representations of the chromosomal distributions of the kiwifruit expansin genes. Centromeric positions are shown according to location of each AcExpansin.

The kiwifruit V3 genome contains 29 chromosomes. To determine the genomic distribution, physical location analysis of 41 expansin genes was performed using the online tool MG2C. Our results showed that 38 AcExpansin genes were unevenly distributed on 22 chromosomes, whereas 3 members of the AcExpansin protein family were attributed to chromosomes that were undetermined (Figure 2). Among the 38 AcExpansin genes, chromosome 3 contained 4 genes; chromosomes 9, 22, and 25 contained 3 genes each; chromosomes 1, 4, 8, 12, 19, 21, and 23 contained 2 genes each; and the other chromosomes contained 1 gene each.

### 2.4. Analysis of Conserved Domains and Gene Structure of the AcExpansin Protein Family

**Figure 3:**
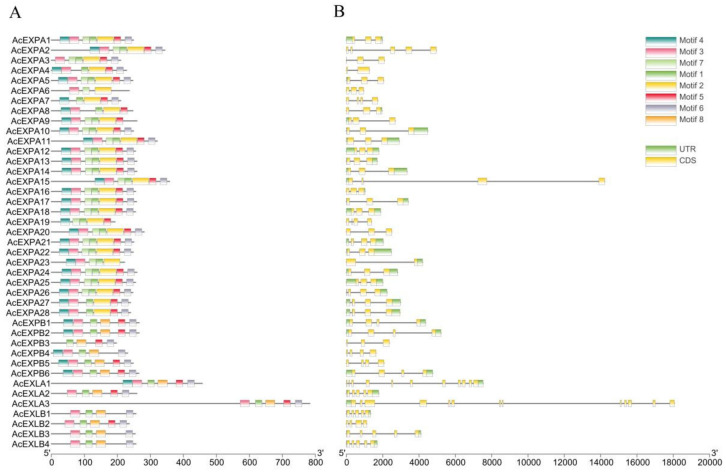
Architectures of the conserved protein motifs and genes of the expansin protein family from kiwifruit. (**A**) MEME motif search results. Conserved motifs are indicated in coloured boxes. (**B**) UTR-CDS structures of the AcExpansin genes. The lengths of UTR and CDS are scaled based on gene length.

In total, eight conserved motifs (named motif 1–8) in AcExpansin proteins were identified using the TBtools software (v.1098696) (Figure 3A). Figure 3 shows that genes from the same subfamily have similar motifs, indicating structural similarities between genes in the same group. Except for motif 8, the other motifs were widely distributed in the AcEXPA subfamily. Motif 2 was only present in the AcEXPA subfamily. Motif 8 was unique to the AcEXPB, AcEXLA, and AcEXLB subfamilies.

The gene structure of the 41 AcExpansin genes was analysed using TBtools and genomic DNA sequences (Figure 3B). Most of the AcEXPA genes included three exons. The number of exons in AcEXPB genes was either three or four. The exon number of AcEXLB genes was either four or five. The exon numbers of the three AcEXLA genes were very different. *AcEXLA1*, *AcEXLA2*, and *AcEXLA3* contained 11, 5, and 13 exons, respectively. 

### 2.5. Intraspecies Collinearity Analysis of Expansion Genes in Kiwifruit

**Figure 4:**
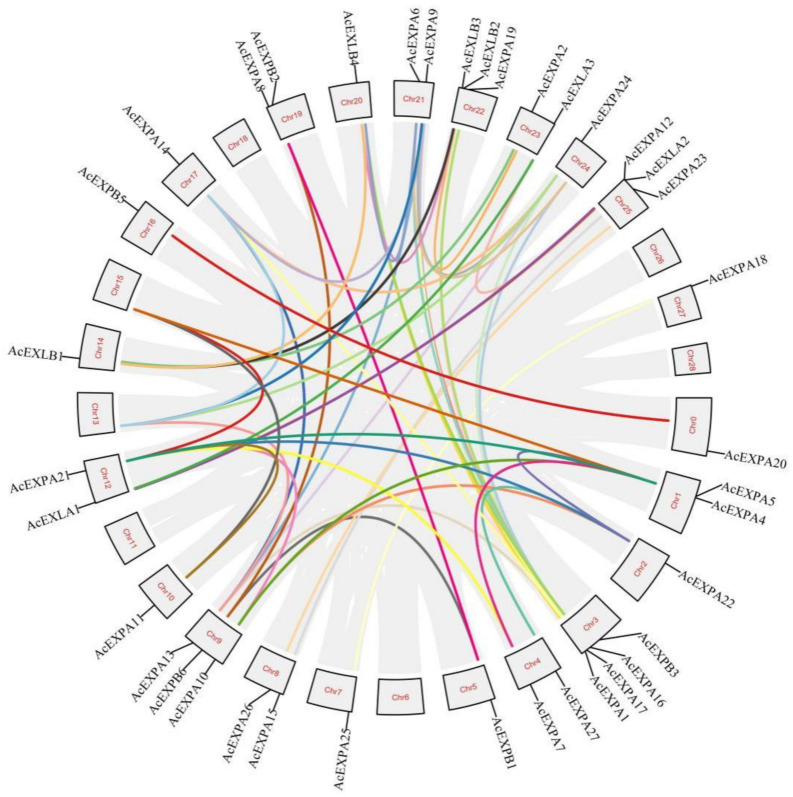
Analysis of collinearity of the expansin genes from kiwifruit. Gray lines in the background indicate collinear blocks within the kiwifruit genome and the lines with different colours highlight syntenic expansin gene pairs.

Tandem and segmental duplication events were identified to investigate gene duplication events. As a result, 43 gene pairs were generated from the 34 segmental duplicated genes (Figure 4). Most segmental duplication genes were found in the EXPA subfamily (*AcEXPA5*, *AcEXPA6*, *AcEXPA9*, *AcEXPA11*, *AcEXPA14*, *AcEXPA16*, *AcEXPA18, AcEXPA21*, *AcEXPA22*, *AcEXPA23, AcEXPA24*, and *AcEXPA28*). Two tandem duplicated genes were identified, forming one pair (Figure 4).

### 2.6. Screening of Candidate Expansin gene under BR and Brassinazole Treatment

**Figure 5:**
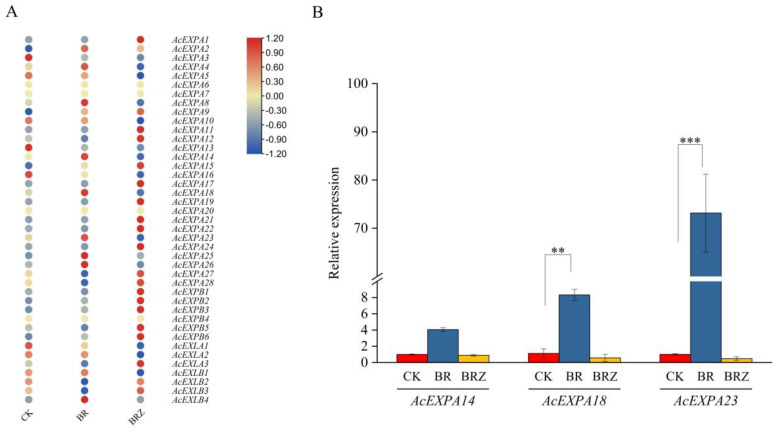
Screening of the candidate gene *AcEXPA23*. (**A**) Heatmap analysis of the expansin protein family genes in kiwifruit. (**B**) Fluorescence quantitative expression of *AcEXPA14*, *AcEXPA18*, and *AcEXPA23*. Asterisks indicate significant differences among treatments (*n* = 3, Fisher’s LSD, ** *p* ≤ 0.05, *** *p* ≤ 0.001).

Based on our previous transcriptome sequencing data (Submission ID: SUB9537634, BioProject ID: PRJNA726005), a heatmap was constructed to analyse the expression patterns of the AcExpansin protein family members with BR and brassinazole treatment (Figure 5A). The results indicated that genes that were upregulated were mainly from the AcEXPA subfamily. Three AcExpansin genes (*AcEXPA14*, *AcEXPA18*, and *AcEXPA23*) were markedly induced by BR and were markedly reduced by brassinazole treatment. We further performed qRT-PCR assays for these three AcExpansin genes, among which *AcEXPA23* had the highest expression level of 78-fold (Figure 5B). Therefore, we selected *AcEXPA23* as a candidate gene to further explore its function in lateral root development.

### 2.7. Subcellular Localisation of AcEXPA23

**Figure 6:**
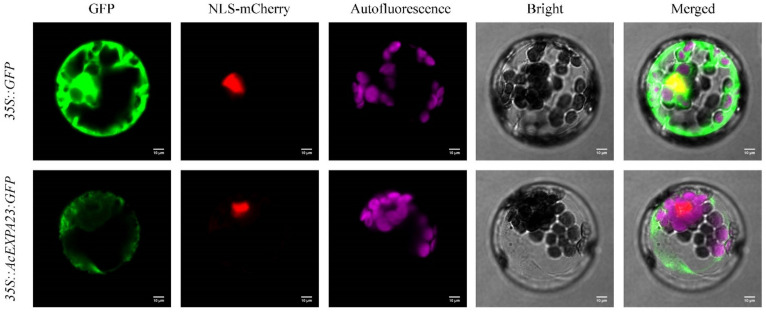
Subcellular localisation of *AcEXPA23*. Vector control (*35S::GFP*) and fusion protein construct *35S::AcEXPA23:GFP* were introduced into the Arabidopsis protoplast.

To determine the subcellular localisation of *AcEXPA23*, we fused the terminator-removed CDS of *AcEXPA23* to green fluorescent protein (GFP) under the control of the CaMV35S constitutive promoter. Using a polyethylene glycol-mediated procedure, *35S::AcEXPA23:GFP* fusion proteins and *35S::GFP* (Control) were transferred into Arabidopsis protoplasts. The results showed that control *35S::GFP* was distributed throughout the whole cell, whereas *35S::AcEXPA23:GFP* was detected in the cytoplasm of the Arabidopsis protoplasts (Figure 6).

### 2.8. Transient Overexpression of AcEXPA23 in Kiwifruit 

**Figure 7:**
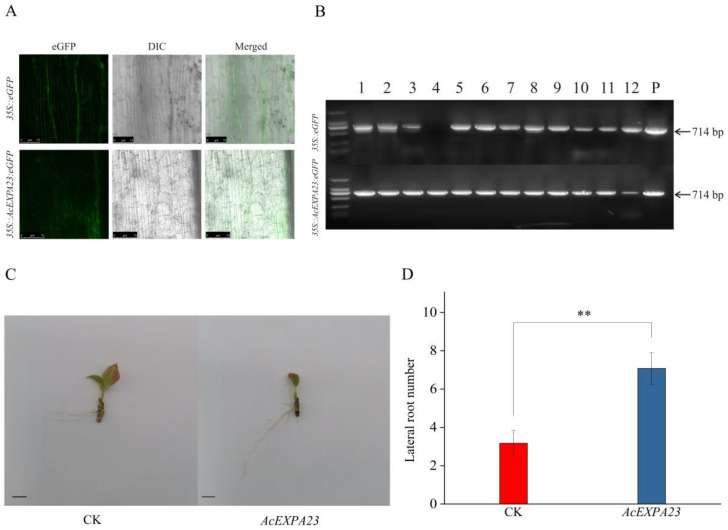
Transient expression of *AcEXPA23* in kiwifruit. (**A**) eGFP signal detection. (**B**) The eGFP sequence was detected by gel electrophoresis. (**C**) Transient expression hairy root phenotype. (**D**) Statistical analysis of the number of lateral roots. Asterisks indicate significant differences among treatments (*n* = 4, Fisher’s LSD, ** *p* ≤ 0.05).

To investigate the role of *AcEXPA23* in lateral root development, transient overexpression was performed in kiwifruit by hairy root infection technology. Laser confocal microscopy was used and a PCR assay was performed to identify positive seedlings (Figure 7A,B). Seedlings in which the fluorescence signal of hairy roots could be observed and amplified from the eGFP sequence fragment were considered as positive plants. Compared with the control, plant overexpression *AcEXPA23* increased by 2.2-times in the number of lateral roots in the hairy roots (Figure 7C,D). The results indicated that *AcEXPA23* plays an important role in the lateral root development of kiwifruit.

### 2.9. AcEXPA23 Overexpression in Kiwifruit Enhanced the Number of Lateral Roots 

**Figure 8:**
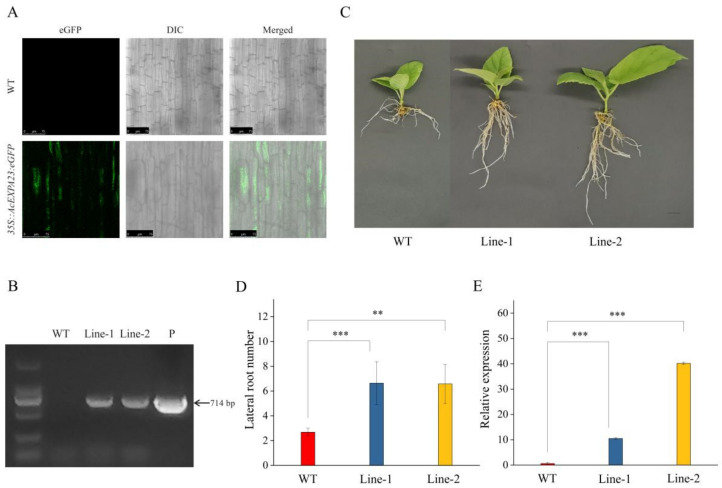
*AcEXPA23* is overexpressed in kiwifruit. (**A**) eGFP signal detection. (**B**) The eGFP sequence was detected by gel electrophoresis. (**C**) Wild-type (WT) and transgenic plant phenotypes. (**D**) Statistical analysis of the number of lateral roots. (**E**) Fluorescence quantitative PCR analysis. Asterisks indicate significant differences among treatments (*n* = 3, Fisher’s LSD, ** *p* ≤ 0.05, *** *p* ≤ 0.001).

To confirm the roles of *AcEXPA23* in kiwifruit, we obtained *AcEXPA23*-overexpressing plants of kiwifruit by transforming explants produced from leaf strips. Similar to transient overexpression, seedlings in which the fluorescence signal of hairy roots was observed and amplified from the eGFP sequence fragment were considered as positive plants (Figure 8A,B). Finally, two overexpression lines were obtained. We observed that overexpression of *AcEXPA23* significantly increased the number of lateral roots compared with that in wild-type kiwifruit (Figure 8C). The number of lateral roots of both Line 1 and Line 2 was 2.45-times higher than that of the wild-type seedlings (Figure 8D). The expression levels of *AcEXPA23* in Line 1 and Line 2 were 17- and 63-times that in wild-type plants, respectively (Figure 8E). Therefore, we conclude that *AcEXPA23* plays an important role in regulating lateral root development in kiwifruit.

## 3. Discussion

The expansin gene is widespread in plants, ranging from algae to higher plants, indicating that the expansin protein family is important for the growth and development of plants [40]. Evolutionary analysis of expansins can provide valuable insights into the regulation of important agronomic traits in kiwifruit genetics and breeding. Our study identified 41 expansin genes in kiwifruit containing two conserved domains, doublepsi-beta barrel and pollen allergen domains, which are characteristic of other expansins identified to date [41]. In kiwifruit, out of the 41 expansins, the number of AcEXPA was dominant compared to the other expansin categories (AcEXPB; AcEXLA and AcEXLB), in line with findings of earlier studies in other species [42,43,44,45,46,47,48,49,50]. Segmental and tandem duplications have been reported to be two of the main causes of gene family expansion in plants [51]. This conclusion is further supported by the finding that most members of the AcExpansin subfamily undergo tandem and segmental duplications. Genes that undergo duplication have three evolutionary outcomes: maintaining the original conserved function, generating new functions, or forming pseudogenes [52]. *AcEXPA1*–*AcEXPA16* are a pair of tandem duplicated genes located on chromosome 3, which are from the EXPA subfamily (Figure 1, Figure 2 and Figure 4). The expression of *AcEXPA1* and *AcEXPA16* was obviously different (Figure 5), indicating that they may have evolved into two genes with different functions. Furthermore, the motif compositions of these two genes were found to be consistent (Figure 3), suggesting that the difference in function may originate from the difference in *cis*-acting elements in the promoter region.

The expression of expansin genes is not only regulated by plant development processes but is also affected by plant hormones. For example, BR induces elongation of excised epicotyl segments and the levels of all EXPA transcripts increased significantly in *Cicer arietinum* [53]. BR promotes *AtEXPA5* expression and positively affects root cell elongation [54]. The transcript level of *EXP1* was upregulated in response to BR treatment in sweet potato [55]. Previously, with heat mapping and qRT-PCR analysis, we also found that BR treatment upregulates *AcEXPA23* expression in kiwifruit roots (Figure 5A) [39]. We analysed the interaction network of *AcEXPA23* using STRING based on AcExpansin orthologues in *Arabidopsis*. This could help us to understand gene function and efficiency [56]. The similarity between *AcEXPA23* and *AtEXPA14* was 83.2%. Studies showed that overexpression of *AtEXPA14* in *Arabidopsis* stimulated the formation of emerged lateral roots, whereas loss of function of *AtEXPA14* reduced auxin-stimulated lateral root formation [18]. We speculate that the *AcEXPA23* gene plays an important role in the regulation of lateral root development.

In this study, transformation experiments demonstrated that overexpression of *AcEXPA23* could significantly enhance the number of lateral roots in kiwifruit (Figure 7 and Figure 8), which was in line with findings of previous studies [18,57,58,59,60]. A recent study on maize yield found that 48% of the yield gain was associated with a decadal climate trend, 39% with agronomic improvements, and only 13% with improvement in genetic yield potential [61]. These findings differed from those of most previous studies, which attributed a much greater weight to genetic yield potential improvement. The continuous activities of human beings have gradually intensified climate change, and it has become more and more important to improve the adaptability of plants [62,63,64]. The present study shows that overexpression of *AcEXPA23* promotes the development of increased lateral roots in kiwifruit. Therefore, BR can be developed into a root regulation product as an improved agronomic measure to improve the root system of kiwifruit, improve the utilisation of water and nutrients, and improve yield and quality.

Expansins are cell-wall-loosening proteins. Most studies showed that expansins are localized in the cell wall, such as *EXPB2* from rice [65], *EXPB2* from *Heterodera avenae* [66], and *EXPA4* from *Chrysanthemum morifolium* [67]. However, some expansins are also reportedly localised to the plasma membrane. For example, the subcellular localisation of epidermal cells in tobacco and onions revealed that *EXPB7* was localised in the plasma membrane of *Hordeum vulgare* [68], *EXPA1* was located in the plasma membrane [69], *EXLA2* was also located in the plasma membrane of tobacco [70]. In the present study, the *35S::AcEXPA23:GFP* fusion protein was transiently expressed in *Arabidopsis* protoplasts and the results showed that the protein was located in the cytoplasm (Figure 6), which may be related to the specific functions of expansins.

## 4. Materials and Methods

### 4.1. Identification of Kiwifruit Expansin Genes 

We identified the expansin protein family genes in kiwifruit using the following four steps. First, the hidden Markov model (HMM) of the two characteristic domains of the expansin gene, pfam01357 (Pollen_allerg_1) and pfam03330 (DPBB_1), from the Pfam database (http://pfam.xfam.org/ (accessed on 4 January 2022)) was downloaded. The expansin protein family genes of kiwifruit were then identified using the Simple HMM Search function in the TBtools software [71]. Second, the *Arabidopsis* expansin protein family protein sequences were downloaded from the TAIR website (www.arabidopsis.org (accessed on 5 January 2022)). The expansin protein family genes of kiwifruit were identified using the Blast Compare Two Seqs function in the TBtools software [71]. Third, the intersection of the genes obtained in the above two steps was considered. Finally, according to the two characteristic domains of the expansin gene, pfam01357 (Pollen_allerg_1) and pfam03330 (DPBB_1), the final screening was performed on the website of InterPro (https://www.ebi.ac.uk/interpro/ (accessed on 6 January 2022)). In addition, the kiwifruit genome protein data used in the first and second identification processes were downloaded from the kiwifruit genome database (http://kiwifruitgenome.org (accessed on 4 January 2022)).

### 4.2. Analysis of AcExpansin Protein Family Characteristics

ClustalW in MEGA11 was used to align the relatedness of AcExpansin protein family gene sequences and a phylogenetic tree was constructed using the neighbour-joining method in MEGA11 (related parameter settings: bootstrap, 1000; model/method, p-distance; gaps/missing data treatment, partial deletion) [72]. Further adjustments and annotations to the evolutionary tree were performed using EVOLVIEW (http://www.evolgenius.info/evolview/#/ (accessed on 16 January 2022)). Chromosome localisation maps were generated online using MG2C (http://mg2c.iask.in/mg2c_v2.1/ (accessed on 21 January 2022)). Motif and gene structures were analysed and visualised using the TBtools software [71]. The physicochemical properties of the proteins were analysed using the online tool Expasy (https://www.expasy.org/ (accessed on 19 January 2022)). Intraspecies collinearity analysis was performed and visualised using the TBtools software [71].

### 4.3. Transient Expression Hairy Root

We performed the following steps for instantaneous conversion. The *AcEXPA23* ORF was first cloned into the pART-CAM-EGFP vector under the control of the CaMV 35S promoter (Table S2). The recombinant vector was then transformed into K599 *Agrobacterium*. The K599 *Agrobacterium* containing the target gene was shaken to make the OD_600_ value reach 0.5–0.8 and the volume of the bacterial solution was approximately 10 mL. The bacterial solution was then centrifuged twice at 8000 rpm for 10 min each and then resuspended in MES buffer (10 mmol/L MES-KOH, pH = 5.2, 10 mmol/L MgCl_2_ and 10 μmol/L acetosyringone). The wild-type kiwifruit (rooted tissue culture seedlings) in good growth condition was selected, and 100–150 µL of bacterial liquid was injected into the young stem with a 0.5 mL syringe, and the infested area is wrapped with degreasing cotton then and placed into the soil. Eighteen plants were used per treatment. After two weeks, the degreasing cotton was removed. Over time, the plants continued to grow. To prevent growing roots from being exposed, they were buried with vermiculite. When hairy roots grew at the infected site and their length was more than 2 cm, the original root system below it was cut off to facilitate rapid growth, and the leaves on the shoots were removed to facilitate survival.

### 4.4. Transformation of A. Chinensis Leaves

The *AcEXPA23* ORF was cloned into the pART-CAM-EGFP vector driven by the CaMV 35S promoter and the recombinant plasmid was subsequently transformed into *A. chinensis* leaves, according to the protocol outlined by Wang [73] (Table S2). Transgenic plants were obtained after approximately 6 months. Transformed plants were identified using PCR and qRT-PCR for the successful verification of transgene incorporation. Two transgenic kiwifruit lines with high *AcEXPA23* mRNA expression were selected for morphological analysis.

### 4.5. Subcellular Localisation

The *AcEXPA23* ORF with a mutated stop codon was cloned between the *Xba*I and *Sal*I sites of the pBI221-GFP vector using T4 DNA ligase (Thermo Scientific, Waltham, MA, USA) (Table S2). The recombinant and control plasmids were transformed into *Arabidopsis* leaf protoplasts as described previously [74]. After 18 h, GFP fluorescence was observed under a laser scanning confocal microscope (FV1000 viewer; Olympus, Tokyo, Japan) at 488 nm with argon-ion laser excitation and GFP was detected at 507 nm. Chloroplast autofluorescence was analysed using 488 nm argon-ion laser excitation, SP 630 nm IR detection, a pinhole of approximately 1.0 units, and an optical section thickness of approximately 0.5 µm.

### 4.6. Quantitative Real-Time Polymerase Chain Reaction

Primer Premier 5 software was used to design qRT–PCR primers for target genes (the primers used are listed in Table S2). The RNA extraction method and qRT-PCR were performed as described by Wu [39]. *Actin* (GenBank EF063572) was used as the normalised control gene [75] (Table S2). Three biological replicates were analysed. The relative expression was calculated using the 2^−ΔΔCt^ method [76].

### 4.7. Statistical Analysis

Excel 2010 (Microsoft Corporation, Redmond, WA, USA), IBM SPSS Statistics 25 (SPSS Inc., Chicago, IL, USA) and Origin 2021 (OriginLab Corporation, Northampton, MA, USA) were used for statistical analyses of the data. Differences between treatments were determined using ANOVA and mean comparisons were made using Fisher’s least significant LSD.

## 5. Conclusions

We identified and analysed the AcExpansin gene family using bioinformatic methods. As such, 41 expansin genes were identified from kiwifruit and classified into four subfamilies, including AcEXPA, AcEXPB, AcEXLA, and AcEXLB. These genes were further analysed for physicochemical properties, chromosomal location, conserved domains, gene structure, and intraspecies collinearity. We found that 41 expansin genes were evolutionarily diverse and conserved at the DNA and protein levels. Finally, we used genetic transformation technology in the kiwifruit to demonstrate that overexpression of *AcEXPA23* can promote the development of increased lateral roots in kiwifruit. This is of great significance for promoting the absorption of water and nutrients in kiwifruit to improve yield and quality.

## Figures and Tables

**Table 1:** Physicochemical characterization of expansin proteins in kiwifruit.

Subfamily	Gene Sequence Number	Gene Name	Number of Amino Acids	Molecular Weight	Theoretical pI	Instability Index	Aliphatic Index	GRAVY
AcEXPA	Actinidia00998	*AcEXPA1*	249	26,626.76	7.53	26.28	67.03	−0.116
	Actinidia02062	*AcEXPA2*	344	37,499.78	9.31	33.13	66.40	−0.074
	Actinidia02869	*AcEXPA3*	210	22,935.07	9.20	24.52	57.67	−0.136
	Actinidia04838	*AcEXPA4*	228	25,123.66	8.59	24.98	73.90	−0.154
	Actinidia04880	*AcEXPA5*	247	26,267.65	9.23	32.29	71.09	−0.023
	Actinidia11513	*AcEXPA6*	236	26,088.61	8.12	22.97	81.48	−0.078
	Actinidia12294	*AcEXPA7*	210	22,278.80	9.72	31.12	67.81	−0.100
	Actinidia12816	*AcEXPA8*	247	27,400.70	10.16	40.00	63.20	−0.431
	Actinidia13883	*AcEXPA9*	259	27,589.34	8.94	41.09	70.42	−0.060
	Actinidia19034	*AcEXPA10*	249	26,683.88	8.88	34.04	64.22	−0.157
	Actinidia19880	*AcEXPA11*	321	34,930.65	9.31	38.12	75.67	0.029
	Actinidia20584	*AcEXPA12*	255	27,582.32	8.01	29.89	73.49	0.014
	Actinidia23935	*AcEXPA13*	259	28,024.18	9.74	35.36	76.45	0.026
	Actinidia26471	*AcEXPA14*	259	28,122.98	9.47	36.04	67.88	−0.066
	Actinidia28781	*AcEXPA15*	358	38,953.08	9.45	46.03	65.98	−0.375
	Actinidia29037	*AcEXPA16*	255	27,616.01	8.89	27.10	64.27	−0.101
	Actinidia29231	*AcEXPA17*	258	27,701.47	9.47	38.78	69.92	−0.006
	Actinidia29762	*AcEXPA18*	255	27,408.12	8.96	26.64	70.78	−0.008
	Actinidia31623	*AcEXPA19*	193	20,731.32	9.30	30.18	64.20	−0.202
	Actinidia32475	*AcEXPA20*	281	31,079.42	9.40	32.99	68.01	−0.441
	Actinidia33207	*AcEXPA21*	250	26,443.57	8.98	33.57	65.56	−0.096
	Actinidia35005	*AcEXPA22*	248	26,471.56	8.38	36.38	61.73	−0.157
	Actinidia35709	*AcEXPA23*	222	23,767.37	8.69	35.10	83.11	0.131
	Actinidia37198	*AcEXPA24*	259	27,821.58	9.39	44.33	70.85	−0.008
	Actinidia37944	*AcEXPA25*	255	27,415.04	9.06	26.35	65.41	−0.074
	Actinidia38621	*AcEXPA26*	247	26,284.66	8.76	28.93	70.32	0.029
	Actinidia38761	*AcEXPA27*	240	25,567.64	8.64	38.18	67.08	−0.049
	Actinidia38779	*AcEXPA28*	240	25,567.64	8.64	38.18	67.08	−0.049
AcEXPB	Actinidia09861	*AcEXPB1*	266	28,632.73	8.79	37.55	77.74	−0.021
	Actinidia11045	*AcEXPB2*	266	28,861.98	8.86	38.09	75.53	−0.064
	Actinidia13599	*AcEXPB3*	197	21,192.05	8.97	37.32	72.79	−0.307
	Actinidia22767	*AcEXPB4*	231	23,757.54	6.21	32.77	57.92	−0.082
	Actinidia33485	*AcEXPB5*	248	25,863.81	4.82	35.78	67.62	−0.119
	Actinidia39310	*AcEXPB6*	265	28,448.37	8.46	33.87	76.87	−0.070
AcEXLA	Actinidia18015	*AcEXLA1*	457	50,895.06	7.04	32.07	81.93	−0.082
	Actinidia20552	*AcEXLA2*	259	28,282.42	8.81	25.72	81.70	0.026
	Actinidia24612	*AcEXLA3*	783	86,023.25	6.88	43.40	82.95	−0.258
AcEXLB	Actinidia10878	*AcEXLB1*	256	27,956.06	4.73	32.56	72.34	−0.200
	Actinidia25907	*AcEXLB2*	236	25,757.25	7.52	30.21	79.28	−0.072
	Actinidia25941	*AcEXLB3*	254	27,840.00	4.81	30.87	67.56	−0.254
	Actinidia31474	*AcEXLB4*	256	28,035.36	5.13	36.77	71.99	−0.186

## Data Availability

The original data for the RNA-seq data were submitted to the SRA database (Submission ID: SUB9537634, BioProject ID: PRJNA726005).

## Supplementary Materials

The following supporting information can be downloaded at: https://www.mdpi.com/article/10.3390/ijms23148026/s1.
